# A New View of the Infection of Scarlet Fever

**Published:** 1823-03-01

**Authors:** 


*A new View of the Infection of Scarlet Fever, illustrated by Remarks on other Contagious Disorders.* By WILLIAM MACMICHAEL, M.D. F.R.S. Fellow of the College of Physicians; Physician Extraordinary to His Royal Highness the Duke of York; and one of the Physicians to the Middlesex Hospital. Octavo, pp. 100. London, Sept. 1822.

THIS little essay is divided into four chapters—the *first* containing, Observations on the improved Value of Life; on the Causes of Epidemic Diseases; on Pellagra; Ergot; and Malaria. The *second* chapter treats of the Contagions of Small-pox, Hydrophobia, Measles, &c. The *third*, is on Scarlet Fever; and the *fourth*, is on the Treatment of the different Forms of Scarlatina. We shall give a brief analysis of these chapters as they stand, interweaving a few observations of our own as we proceed.

*Chap.* I. It is an interesting fact, now well established, that the ratio of mortality in England, has decreased nearly one third within the last forty years. In 1780, the calculation was 1 in 40 annually; whereas, it is evident, from the population returns to Parliament, that the annual mortality is now only one in fifty-eight. This change may very fairly be attributed to the bettered condition of the poor—the increase of temperance in all classes—the disappearance or mitigation of many fatal diseases—vaccination—improved medical practice. But, while the sum total of human affliction has been reduced, there is considerable disparity of reduction in the different items. Thus, rickets and scrophula, according to Dr. M. have decreased, while gout, consumption, palsy, and mania, have increased in fatality. Certain forms of scrophula and rickets may have been reduced in number, from the greater cleanliness and better diet of the poor; but, as we consider the prominent character of phthisis to be scrofulous tubercles in the lungs, it may be questioned whether scrophula, on the whole, has declined. On the other hand, we are doubtful that gout is increasing. Or, if it be, how are we to reconcile this with the acknowledged increase of temperance in all ranks of society?* That the great class of the NEUROSES, with their host of consecutive evils have, for several years past, been on the advance, there can be no doubt. The wide spread of intellectual excitement—the revolutions, political and moral, with which Europe has been torn—the sudden elevations and depressions of fortune—the anxiety and perturbation attending a hazardous and excessive spirit of mercantile speculation—the fanaticism of some, and the atheism of others, (for extremes approximate,) may explain the greater prevalence of mental diseases now, than formerly. The increase of palsy, our author attributes to "the less general use of blood-letting" now than formerly—especially at spring and fall, when our forefathers regularly got blooded. That something may be attributed to this last cause we are not inclined to deny; but we are far from considering it as the principal cause. The circumstances which we have pointed out as predisposing to maniacal and other affections of the brain and nervous system, will equally conduce to the production of paralytic and apoplectic complaints.†

---

* Our author has surely been rather inconsistent, when, at page 2, he alludes to the prevalence of temperate habits, "throughout all orders of society," and then, at page 3, speaks of the *indulgences and vices* of civilized life, as accounting for the increase of gout. If the latter be increasing, which we doubt, it must, we believe, be owing to sedentary habits and mental anxiety operating on the digestive organs, and through their derangements, inducing gout and other diseases.

† Dr. Dovar, (inventor of the P. Ipecac. comp.) who, by the way, was one of the most canting old quacks that ever perpetuated his own disgrace by becoming an author, states, in his Physician's Legacy, that

Our author properly remarks, that the more obvious causes of those disorders which affect great numbers of people at the same time, in other words, of epidemics, must be sought for in unwholesome or defective diet—noxious exhalations from the earth—human contagion—and atmospheric influence. We agree with Dr. Macmichael, that improvements in agriculture, by furnishing a greater abundance of the necessaries of life, counteract the first of these causes; and, that quarantine has probably kept our shores free from visitations of plague; but, we are disposed to think, that plague would make little progress, now-a-days, in England, even were it regularly imported to this island. Although, therefore, we should not vote for the total abrogation of quarantine laws, we attach far less importance to them than our author, and we hope to see them greatly modified, even in our own time.

"The *universal* practice of vaccination, (says our author,) would certainly completely exterminate the small-pox." This we doubt, though we are unequivocal advocates for vaccination; and strenuously recommend its *universal* adoption, if possible.

PELLAGRA.

Several accounts of this curious disease have been published in England; but it has not hitherto been noticed in this Journal. We shall give some short account of it here. It has been principally observed in those provinces of Italy, lying between the Alps and the Po. It is a cutaneous affection, confined to the peasants employed in the cultivation of the soil; and, especially, in the raising of the vine, maize, rice, and millet.

"In the early stage of the complaint, red spots, with slight elevations of the cuticle, resembling lepra, are observable; the skin becomes dry and scaly; vague and irregular pains are felt; and in its inveterate form, the disease assumes an appearance not unlike Icthyosis. The malady then abates; but as the summer of the following year approaches, it recurs with increased violence; spasms, anxiety, depression of spirits, cachexy, idiotcy, and mania are the last symptoms." 12.

The peasants abovementioned, live in extreme misery, not-

---

during an extensive practice of forty-seven years duration, he never met with but two cases of apoplexy. This was in the beginning of last century.

We may here mention, that the commentator on Dr. Dovar's Legacy states, his having cured epilepsy several times by the misletoe of the oak, ℈j. thrice a day. We believe that the remedy is not generally considered as having been discovered so long ago as one hundred years, for this particular complaint.

withstanding the great fertility of those provinces—having little animal food, and their bread (of maize) being ill-fermented and deficient in salt. The cure, as may easily be supposed, consists of generous food, wine, tonics, and the warm bath.

### ERGOT.

Rye is subject to a disease called Ergot—in English horned or spurred rye. Bread made of it has a nauseous acid taste, and has been thought to be the cause of a spasmodic and gangrenous disorder.

This epidemic, which was also called the *ignis sacer*, raged in Sologne about the year 1650, but was probably owing more to starvation than to the use of injured corn. The disease was accompanied with lassitude, debility, torpor, swelling, and sense of burning heat and excruciating pain in the lower limbs, which became shrivelled, and at length gangrenous. Another disorder ascribed to the ergot, was attended with general convulsions of the muscles. It was from this circumstance, we believe, that ergot was first tried to excite uterine action by one of our continental or American brethren.

### MALARIA.

Marsh effluvia, our author properly enough thinks, is too confined a term for Malaria, since there are many marshy districts where ague does not prevail; while, on the other hand, intermittents and fevers of that type are often seen, though the soil be dry, and the ground elevated. There are very few places, we believe, however, of this last description, that prove to be malarious, unless they are in the vicinity of some marsh, or unless they consist of a bibulous soil that quickly absorbs the moisture, and afterwards gives it out to the action of the sun, imbued with decomposed animal and vegetable matter. Of the exact nature indeed of this invisible agent termed malaria, or vegeto-animal miasma, we are entirely ignorant. Its effects are very conspicuous in several parts of Italy, especially in that part called the Maremma, a district that stretches from Leghorn to Terracina, varying in breadth from thirty to forty miles, and being in length about 192 geographical miles, lying near the sea. The greater number of places in this line are dry, airy, and elevated; nor does the victim perceive any visible sign of the presence of the destructive poison he is inhaling, "for the tranquillity of the air and the freshness of the verdure around him, would lead him to suppose he was in the most healthy region." Even in such places, there can be no doubt that the deleterious material, whatever be its composition, must issue from the earth. We all know that the atmosphere preserves its general constituents under all circumstances, and in all kinds of locality; it is only from the earth that contamiation can arise, however it may float about afterwards through the medium of the air.

That some cause operating under ground is productive of malaria in Italy, is, we think, fairly indicated by its gradual and progressive march in certain directions—thus it every year comes closer to, and reaches some part of Rome where it was before unknown.

### Chap. II.—*Contagion.*

How the contagions of small-pox, hydrophobia, measles, plague, &c. first originated, Dr. Macmichael does not attempt to explain. He thinks they existed anterior to any tradition or historical record—that they were perpetuated from age to age and from year to year—that they were confined in the first instance, to some remote district, and were gradually disseminated over the earth by war, conquests, political revolutions, commercial intercourse, and the accidental visits of travellers.* This appears to us to be viewing contagions as plants or animals, which never can arise from any cause or combination of causes except specific propagation. For our own parts we think it far more likely that the same causes which produced these contagions in the *first* instance, may have many times since done the same thing—and that this is the case even to the present time, in several of the contagions, as hydrophobia, measles, scarlatina, &c.

Our author, for instance, thinks that hydrophobia can only be produced "by communication from one rabid animal to another." That it has no relation to the heat of the weather is proved, he thinks, by the disease being prevalent in Russia, and *unknown in India.*† As to Russia we cannot say; but there is no part of the world where hydrophobia is more prevalent than in India. Can Dr. Macmichael have forgotten the great noise which the practice of blood-letting in hydrophobia, first instituted in India by Messrs. Tymon and Shoolbred, made among the profession here?‡ We cannot understand Dr. Macmichael's reasoning in the same page, where he proposes the laws of "strict quarantine" against hydrophobia.§

---

* P. 24, 25. † P. 27. ‡ Vide Ed. Journal, vol. 9.

§ Lest we may be supposed to misrepresent Dr. Macmichael, for which we should be extremely sorry, we shall give the passage in his own words.

The following sentence does not correspond with our observations and experience.

"Parents considering the measles as a disease almost inevitable, have wisely chosen to expose their children to the contagion at such auspicious times; (when they are epidemical and mild;) so that the disorder may be once well over, and all further anxiety at an end."

We firmly believe that parents would be wise in doing so, especially *in mild seasons of the year*, for surely these must be the great modifying causes; but we have very rarely seen this wisdom manifested by parents. They generally like to put off the evil hour as long as possible, in this and in most other of the eruptive diseases.

On the question why contagious diseases at one time assume a mild type, and at another appear under an alarming aspect, our author offers some ingenious remarks, which we are very far from thinking quite hypothetical or devoid of solid foundation. Dr. M's explanation is this:—that instead of an epidemic constitution of the *air*, there is an epidemic constitution of the human system prevailing at the time. But he shall speak for himself.

"In the absence of all other explanation, it has generally been agreed to attribute this difference of type to some occult quality of the air; for we are not more advanced now in our knowledge of the subject, than they were in the days of Sydenham. The *constitutio aeris* is still appealed to as necessary, if not to generate, at least to cause the universal diffusion of contagious fever. But if instead of employing so vague and unmeaning a term, we were content to speak of a *constitutio epidemica*, or that peculiar state or condition of body, into which a great number of people are brought by having been subjected to the operation of the same physical and moral causes, something more distinct and intelligible would be expressed.

"These causes are very numerous, and various in their character; as previous hot, cold, or damp weather, deficient or bad diet, fatigue, grief, anxiety, &c. Being all similarly predisposed, it is not strange that if a number of people should fall into the same dis-

---

"If the opinion that this poison, like the other infections, is the result of an original unextinguished contagion, be correct, does it not point out a simple method of extinguishing it in this country, viz. by adopting measures similar to those of the strict quarantine, to which we are indebted for our present exemption from the horrors of the plague?" P. 27.

In what possible way, we ask, could the laws of quarantine be put in execution against mad dogs? Before they become mad we know nothing of the business, and afterwards they generally run away.

ease, they should have it in the same way, whether mild or severe, in other words, that an *epidemic constitution* should prevail." 32.

The only remark we shall make on this explanation is—that as this prevailing constitution must have been produced by the physical agents around us, so it seems to matter little whether these agents modify the constitution, the contagion remaining the same—or modify the contagion the constitution remaining the same. Is it not probable that both the contagion and the constitution are modified by the surrounding agents abovementioned ?* We have no direct proof for or against either position.

Dr. Macmichael's observations on the contagion of typhus fever need not detain us. We do not see any thing new in them. We cannot agree with him in his opinion that "typhus fever does not now *originate* in any individual." As a proof of this Dr. M. brings forward two passages from Dr. Lind. The first was an *on dit* of the celebrated doctor. The surgeon of the Panther told him that during the prevalence of scurvy on board that ship the sick birth was dreadfully crowded, but that no contagious fever was generated. Such might be the case. We know that the great prevalence of one disease sometimes—indeed generally, precludes the appearance of others.

The converse of the above—viz. the proof of imported contagion is thus stated :—

"The company of the Loestoffe were in perfect health during the eight months they were in America, and until a few days before their departure from Quebec. At that time six recovered marines came on board from *Point Levi* hospital, and *in forty eight hours afterwards, among her company of two hundred people, fifty were seized with fevers and fluxes!* In some the sickness began with a flux, in others with a fever; but the flux was generally moderate and gentle. The fever continued commonly from five to ten days; two patients were distressed with it for a whole month." 44.

We ask Dr. M. whether it coincides with his experience of typhus contagion, that it produces fever in forty-eight hours after exposure to it—and that when conveyed through the medium of recovered men ? If so, it is contrary to our experience. The whole of Dr. Lind's statement is founded on that most fallacious kind of evidence—*post hoc ergo propter hoc*. If the six marines had not come on board 48

---

* This seems to have been the opinion of Dr. W. Heberden, who remarks, "That the presence of infectious matter is not alone sufficient to make the plague epidemical, but that some concurrent state of the air, *and of the human body*, is likewise necessary."—*Increase and Decrease of Diseases, p.* 95.

hours before, the fevers and fluxes would have equally appeared. Their causes were longer in operation than two days; but it was far easier for the surgeon to place the whole to the account of the six marines, than to investigate the etiology of the fever and flux. We have seen enough of these short cuts to the origin of diseases to make us very indifferent to such *on dits* as the above tale of the Panther.

### Chap. III.—*Scarlet Fever.*

It is in this chapter that the jet of our author's essay comes out in form.

Scarlatina, Dr. M. observes, is so various in its character, and one form of it is so extremely mild, "*that it does most certainly pass through the system unobserved;* and in many more instances under the name of a *rash*, exciting no alarm." The position which we have marked in italics is meant, we believe, *first*, to account for the number of people who think they have never had scarlatina; and *secondly*, to take away the excuse for not putting children in the way of the contagion, from the vain hope of escaping the disease altogether. Our author, therefore, assumes that scarlatina is as general in affecting the human constitution as small-pox or measles—its non-perceptibility, in some of its forms, accounting for the immunity which some people *appear* to have possessed.* But in the first place, we fear, Dr. Macmichael can offer us no *proof* that those who have passed through life without the *ostensible* forms of scarlet fever must yet have had the *imperceptible* form of the disease. In the second place, can Dr. M. assure us that these imperceptible forms, or even the visible *rashes* will certainly secure us against the more dangerous forms of the disease? We fear he cannot offer us any positive assurance on either of these points; and if he cannot, we imagine that parents will be unwilling to expose their children to the disease, however mild it may be at the time. Again, can we be sure that because the epidemic is mild, generally, it will be equally so in individual cases? We apprehend not. At least we have seen some children in the same family have the disease in the slightest manner, while others had it in the most terrible form, where the source of the contagion was the same in both cases.†

---

* "Adults," says Dr. Willan, "are not very susceptible of the contagion (of scarlatina.) Several physicians and apothecaries of London have never felt its effects, notwithstanding their attendance on many hundreds in the disease, often under the most unfavourable circumstances."—*On Cutaneous Diseases.*

† Willan, though he acknowledges that each epidemic has a reigning

Notwithstanding the objections which we have stated, we are disposed to think that could Dr. Macmichael's proposal be put fully and universally into practice, there would be considerably less mortality and ill consequences from scarlatina than there now are—because there would be a *greater chance* of a mild disease in a favourable season and epidemic, than in contrary circumstances. But although this would be the general or average result, still as individual events would now and then be disastrous, people will never be persuaded to run the risk, lest they should happen to be the victims.*

In giving the following recapitulation in the author's own words, we take our leave of him with every sentiment of respect and esteem.

"To recapitulate the substance of the foregoing observations, I have to remark, that if they are founded in truth, and warranted by daily experience, we must come to the following conclusions.

"As the causes producing epidemic diseases are not very numerous, and are much subject to our own control, the means of lengthening the duration and increasing the comforts of life are to a great extent placed in our own hands.

"It has been seen that by a strict enforcement of the laws of quarantine, we have been able to banish the plague, and by the employment of vaccination, we have it in our power (if we choose to avail ourselves to the utmost of the benefits of that great discovery) to exterminate the small-pox; a contagion still more fatal than the plague itself. For the latter disease has never yet been known in India, China, North or South America, nor in the arctic or tropical regions; while the small-pox has spared no nation, but has made its appearance in all seasons, and extended its ravages over every climate of the earth.

"Till we are happily enabled (and the hope ought not to be treated as wild and chimerical, for who would, a few years ago, nave believed in the possibility of a discovery so beneficial as vaccination?) to extinguish the other contagions, we should watch the favourable types of these disorders, and court rather than avoid their

---

character very generally conspicuous, yet he adduces as proof that the simple scarlatina, the S. anginosa and S. maligna all proceed from the same source, the following fact—"because under the same roof, in large families, some individuals have the disease in one form, some in another, *about the same period*."—*On Cutaneous Diseases*, p. 281. If this be the case, what security is there of having a mild disease at any one given time by exposure to the contagion?

* Sir Gilbert Blane, in his recently published select dissertations, states as follows:—"One attack of scarlet fever *generally* secures from a future one; but I know a case of a young lady who had it distinctly three times." 210.

infection at such auspicious periods. For though it be true that some few persons pass through the vicissitudes of a long life, without ever catching the scarlet fever, such an occurrence is by no means common. I have endeavoured to show that its frequency has been, from carelessness and inattention, greatly overrated.

"The chance, therefore, of such an escape, ought to have no weight in the calculations of prudence and sound reasoning, as in the course of their lives, it is probable, that nine persons out of ten have undergone scarlet fever, in various degrees and shades of violence, from the unnoticed rash to the virulent form of putrid sore throat.

There is one other remark which occurs to me before I conclude, from which much practical benefit may occasionally be derived. Whenever it happens that a person infected with typhus fever, or any other contagious disease of a malignant character, is necessarily confined in a house occupied by a numerous family, he should be removed to the upper story. The current of heated air is naturally upwards, and the atmosphere loaded with the contagious steams, emanating from the patient's body, will (if he be in a lower apartment) diffuse themselves over the whole house, whereas, if he be placed above, they will have a ready and immediate vent." 100.

